# Impacts of Waste from Concentrated Animal Feeding Operations on Water Quality

**DOI:** 10.1289/ehp.8839

**Published:** 2006-11-14

**Authors:** JoAnn Burkholder, Bob Libra, Peter Weyer, Susan Heathcote, Dana Kolpin, Peter S. Thorne, Michael Wichman

**Affiliations:** 1 North Carolina State University, Raleigh, North Carolina, USA; 2 Iowa Geological Survey, Iowa City, Iowa, USA; 3 The University of Iowa, Iowa City, Iowa, USA; 4 Iowa Environmental Council, Des Moines, Iowa, USA; 5 Toxic Substances Hydrology Program, U.S. Geological Survey, Iowa City, Iowa, USA; 6 University Hygienic Laboratory, Iowa City, Iowa, USA

**Keywords:** ecology, human health, poultry, swine, water contaminants, wildlife

## Abstract

Waste from agricultural livestock operations has been a long-standing concern with respect to contamination of water resources, particularly in terms of nutrient pollution. However, the recent growth of concentrated animal feeding operations (CAFOs) presents a greater risk to water quality because of both the increased volume of waste and to contaminants that may be present (e.g., antibiotics and other veterinary drugs) that may have both environmental and public health importance. Based on available data, generally accepted livestock waste management practices do not adequately or effectively protect water resources from contamination with excessive nutrients, microbial pathogens, and pharmaceuticals present in the waste. Impacts on surface water sources and wildlife have been documented in many agricultural areas in the United States. Potential impacts on human and environmental health from long-term inadvertent exposure to water contaminated with pharmaceuticals and other compounds are a growing public concern. This work-group, which is part of the Conference on Environmental Health Impacts of Concentrated Animal Feeding Operations: Anticipating Hazards—Searching for Solutions, identified needs for rigorous ecosystem monitoring in the vicinity of CAFOs and for improved characterization of major toxicants affecting the environment and human health. Last, there is a need to promote and enforce best practices to minimize inputs of nutrients and toxicants from CAFOs into freshwater and marine ecosystems.

## Background and Recent Developments

### Concentrated animal feed operations and water quality

Animal cultivation in the United States produces 133 million tons of manure per year (on a dry weight basis) representing 13-fold more solid waste than human sanitary waste production [U.S. Environmental Protection Agency (U.S. EPA) 1998]. Since the 1950s (poultry) and the 1970s–1980s (cattle, swine), most animals are now produced for human consumption in concentrated animal feeding operations (CAFOs). In these industrialized operations, the animals are held throughout their lives at high densities in indoor stalls until they are transported to processing plants for slaughter. There is substantial documentation of major, ongoing impacts on aquatic resources from CAFOs, but many gaps in understanding remain.

### Contaminants detected in waste and risk of water contamination

Contaminants from animal wastes can enter the environment through pathways such as through leakage from poorly constructed manure lagoons, or during major precipitation events resulting in either overflow of lagoons and runoff from recent applications of waste to farm fields, or atmospheric deposition followed by dry or wet fallout (Aneja 2003). The magnitude and direction of transport depend on factors such as soil properties, contaminant properties, hydraulic loading characteristics, and crop management practices (Huddleston 1996). Many contaminants are present in livestock wastes, including nutrients (Jongbloed and Lenis 1998), pathogens (Gerba and Smith 2005; Schets et al. 2005), veterinary pharmaceuticals (Boxall et al. 2003; Campagnolo et al. 2002; Meyer 2004), heavy metals [especially zinc and copper; e.g., Barker and Zublena (1995); University of Iowa and Iowa State Study Group (2002)], and naturally excreted hormones (Hanselman et al. 2003; Raman et al. 2004). Antibiotics are used extensively not only to treat or prevent microbial infection in animals (Kummerer 2004), but are also commonly used to promote more rapid growth in livestock (Cromwell 2002; Gaskins et al. 2002; Liu et al. 2005). In addition, pesticides such as dithiocarbamates are applied to sprayfields (Extension Toxicology Network 2003). Although anaerobic digestion of wastes in surface storage lagoons can effectively reduce or destroy many pathogens, substantial remaining densities of microbial pathogens in waste spills and seepage can contaminate receiving surface- and ground-waters (e.g., Burkholder et al. 1997; Mallin 2000). Pharmaceuticals can remain present as parent compounds or degradates in manure and leachates even during prolonged storage. Improper disposal of animal carcasses and abandoned livestock facilities can also contribute to water quality problems. Siting of livestock operations in areas prone to flooding or where there is a shallow water table increases the potential for environmental contamination.

The nutrient content of the wastes can be a desirable factor for land application as fertilizer for row crops, but overapplication of livestock wastes can overload soils with both macronutrients such as nitrogen (N) and phosphorous (P), and heavy metals added to feed as micronutrients (e.g., Barker and Zublena 1995). Overapplication of animal wastes or application of animal wastes to saturated soils can also cause contaminants to move into receiving waters through runoff and to leach through permeable soils to vulnerable aquifers. Importantly, this may happen even at recommended application rates. As examples, Westerman et al. (1995) found 3–6 mg nitrate (NO_3_)/L in surface runoff from sprayfields that received swine effluent at recommended rates; Stone et al. (1995) measured 6–8 mg total inorganic N/L and 0.7–1.3 mg P/L in a stream adjacent to swine effluent sprayfields. Evans et al. (1984) reported 7–30 mg NO_3_/L in subsurface flow draining a sprayfield for swine wastes, applied at recommended rates. Ham and DeSutter (2000) described export rates of up to 0.52 kg ammonium m^−2^ year^−1^ from lagoon seepage; Huffman and Westerman (1995) reported that groundwater near swine waste lagoons averaged 143 mg inorganic N/L, and estimated export rates at 4.5 kg inorganic N/day. Thus, nutrient losses into receiving waters can be excessive relative to levels (~ 100–200 μg inorganic N or P/L) known to support noxious algal blooms (Mallin 2000). In addition to contaminant chemical properties, soil properties and climatic conditions can affect transport of contaminants. For example, sandy, well-drained soils are most vulnerable to transport of nutrients to underlying groundwater (Mueller et al. 1995). Nutrients can also readily move through soils under wet conditions (McGechan et al. 2005).

### Presence of contaminants in water sources

The presence of many contaminants from livestock waste has been documented in both surface water and groundwater supplies in agricultural areas within the United States (e.g., Campagnolo et al. 2002; Kolpin et al. 2002; Meyer 2004). Urban wastewater streams also contain these contaminants, and efforts to accurately determine sources of contamination are under way (Barnes et al. 2004; Cordy et al. 2004; Kolpin DW, unpublished data). The U.S. Geological Survey (USGS) began pilot surveillance programs for organic wastewater contaminants in 1999 and expanded that effort to a national scale over the past 5 years (Kolpin et al. 2002). Recent USGS efforts have focused specifically on water quality in agricultural locations (Kolpin DW, unpublished data). Nutrient levels have been detected in high parts per million (milligrams per liter) levels; pharmaceuticals and other compounds are generally measured in low levels (ppb [micrograms per liter]). In Europe, surveillance efforts conducted in Germany documented the presence of veterinary pharmaceuticals in water resources (Hirsch et al. 1999).

Animal wastes are also rich in organics and high in biochemical oxygen-demanding materials (BOD); for example, treated human sewage contains 20–60 mg BOD/L, raw sewage contains 300–400 mg BOD/L, and swine waste slurry contains 20,000–30,000 mg BOD/L (Webb and Archer 1994). Animal wastes also carry parasites, viruses, and bacteria as high as 1 billion/g (U.S. EPA 1998). Swine wastes contain > 100 microbial pathogens that can cause human illness and disease [see review in Burkholder et al. (1997)]. About one-third of the antibiotics used in the United States each year is routinely added to animal feed to increase growth (Mellon et al. 2001). This practice is promoting increased antibiotic resistance among the microbial populations present and, potentially, increased resistance of naturally occurring pathogens in surface waters that receive a portion of the wastes.

### Contaminant impacts

Some contaminants pose risks for adverse health impacts in wildlife or humans. The effects of numerous waterborne pathogens on humans are well known, although little is known about potential impacts of such microorganisms on aquatic life. With respect to nutrients, excessive phosphorus levels can contribute to algal blooms and cyanobacterial growth in surface waters used for recreation and as sources of drinking water. Research is beginning to investigate the environmental effects, including endocrine disruption and antibiotic resistance issues (Burnison et al. 2003; Delepee et al. 2004; Fernandez et al. 2004; Halling-Sorensen et al. 2003; Sengelov et al. 2003; Soto et al. 2004; Wollenberger et al. 2000). However, knowledge is limited in several crucial areas. These areas include information on metabolites or environmental degradates of some parent compounds; the environmental persistence, fate, and transport and toxicity of metabolites or degradates (Boxall et al. 2004); the potential synergistic effects of various mixtures of contaminants on target organisms (Sumpter and Johnson 2005); and the potential transport and effects from natural and synthetic hormones (Hanselman et al. 2003; Soto et al. 2004). Further, limited monitoring has been conducted of ecosystem health in proximity to CAFOs, including monitoring the effects on habitats from lagoon spills during catastrophic flooding (Burkholder et al. 1997; Mallin et al. 1997; Mallin et al. 2000).

### Ecologic and wildlife impacts

Anoxic conditions and extremely high concentrations of ammonium, total phosphorus, suspended solids, and fecal coliform bacteria throughout the water column for approximately 30 km downstream from the point of entry have been documented as impacts of waste effluent spills from CAFOs (Burkholder et al. 1997; Mallin et al. 2000). Pathogenic microorgan-isms such as *Clostridium perfringens* have been documented at high densities in receiving surface waters following CAFO waste spills (Burkholder et al. 1997). These degraded conditions, especially the associated hypoxia/anoxia and high ammonia, have caused major kills of freshwater fish of all species in the affected areas, from minnows and gar to largemouth bass, and estuarine fish, including striped bass and flounder (Burkholder et al. 1997). Waste effluent spills also stimulated blooms of toxic and noxious algae. In freshwaters, these blooms include toxic and noxious cyanobacteria while in estuaries, harmful haptophytes and toxic dinoflagellates arise. Most states monitor only water-column fecal coliform densities to assess whether waterways are safe for human contact. World Health Organization (WHO) guidelines for cyanobacteria in recreational water are 20,000 cyanobacterial cells/mL, which indicates low probability of adverse health effects, and 100,000 cyanobacterial cells/mL, which indicates moderate probability of adverse health effects (WHO 2003). Yet fecal bacteria and other pathogenic microorganisms typically settle out to the sediments where they can thrive at high densities for weeks to months following CAFO waste effluent spills (Burkholder et al. 1997).

The impacts from CAFO pollutant loadings to direct runoff are more substantial after such major effluent spills or when CAFOs are flooded and in direct contact with surface waters (Wing et al. 2002). Although the acute impacts are often clearly visible—dead fish floating on the water surface, or algal overgrowth and rotting biomass—the chronic, insidious, long-term impacts of commonly accepted practices of CAFO waste management on receiving aquatic ecosystems are also significant (U.S. EPA 1998). One purpose of manure storage basins is to reduce the N content of the manure through volatilization of ammonia and other N-containing molecules. Many studies have shown, for example, that high nutrient concentrations (e.g., ammonia from swine CAFOs, or ammonia oxidized to NO_3_, or phosphorus from poultry CAFOs) commonly move off-site to contaminate the overlying air and/or adjacent surface and sub-surface waters (Aneja et al. 2003; Evans et al. 1984; Sharpe and Harper 1997; Sharpley and Moyer 2000; Stone et al. 1995; U.S. EPA 1998; Webb and Archer 1994; Westerman et al. 1995; Zahn et al. 1997). Inorganic N forms are added to the atmosphere during spray practices, and both ammonia and phosphate can also adsorb to fine particles (dust) that can be airborne. The atmospheric depositions are noteworthy, considering that a signifi-cant proportion of the total ammonium from uncovered swine effluent lagoons and effluent spraying (an accepted practice in some states) reenters surface waters as local precipitation or through dry fallout (Aneja et al. 2003; U.S. EPA 1998, 2000). The contributed nutrient concentrations from the effluent greatly exceed the minimal levels that have been shown to promote noxious algal blooms (Mallin 2000) and depress the growth of desirable aquatic habitat species (Burkholder et al. 1992). The resulting chronically degraded conditions of nutrient overenrichment, while not as extreme as during a major waste spill, stimulate algal blooms and long-term shifts in phytoplankton community structure from desirable species (e.g., diatoms) to noxious species.

A summary of the findings from a national workshop on environmental impacts of CAFOs a decade ago stated that there was “a surprising lack of information about environmental impacts of CAFOs to adjacent lands and receiving waters” (Thu K, Donham K, unpublished data). Although the knowledge base has expanded since that time, especially regarding adverse effects of inorganic N and P overenrichment and anoxia, impacts of many CAFO pollutants on receiving aquatic ecosystems remain poorly understood. As examples, there is poor understanding of the impacts of fecal bacteria and other microbial pathogens from CAFO waste effluent contamination on aquatic communities; impacts of antibiotic-resistant bacteria created from CAFO wastes on aquatic life; impacts of organic nutrient forms preferred by certain noxious plankton; impacts from the contributed pesticides and heavy metals; and impacts from these pollutants acting in concert, additively or synergistically. This lack of information represents a critical gap in our present ability to assess the full extent of CAFO impacts on aquatic natural resources.

Despite their widespread use, antibiotics have only recently received attention as environmental contaminants. Most antibiotics are designed to be quickly excreted from the treated organism. Thus, it is not surprising that antibiotics are commonly found in human and animal waste (Christian et al. 2003; Dietze et al. 2005; Glassmeyer et al. 2005; Meyer 2004) and in water resources affected by sources of waste (Glassmeyer et al. 2005; Kolpin et al. 2002). Although some research has been conducted on the environmental effects from antibiotics (e.g., Brain et al. 2005; Jensen et al. 2003), much is yet to be understood pertaining to long-term exposures to low levels of antibiotics (both individually and as part of complex mixtures of organic contaminants in the environment). The greatest risks appear to be related to antibiotic resistance (Khachatourians 1998; Kummerer 2004) and natural ecosystem functions such as soil microbial activity and bacterial denitrification (Costanzo et al. 2005; Thiele-Bruhn and Beck 2005).

### Human health impacts

Exposure to waterborne contaminants can result from both recreational use of affected surface water and from ingestion of drinking water derived from either contaminated surface water or groundwater. High-risk populations are generally the very young, the elderly, pregnant women, and immunocompromised individuals. Recreational exposures and illnesses include accidental ingestion of contaminated water that may result in diarrhea or other gastrointestinal tract distress from waterborne pathogens, and dermal contact during swimming that may cause skin, eye, or ear infections. Drinking water exposures to pathogens could occur in vulnerable private wells; under normal circumstances community water utilities disinfect water sufficiently before distribution to customers. Cyanobacteria (blue–green algae) in surface water can produce toxins (e.g., microcystins) that are known neuro-toxins and hepatotoxins. Acute and chronic health impacts from these toxins can occur from exposures to both raw water and treated water (Carmichael et al. 2001; Rao et al. 2002). Removal of cyanotoxins during drinking water treatment is a high priority for the drinking water industry (Hitzfield et al. 2000; Rapala et al. 2002). The WHO has set a provisional drinking water guideline of 1 μg microcystin-LR/L (Chorus and Bartram 1999). While there are no drinking water standards in the United States for cyanobacte-ria, they are on the U.S. EPA Unregulated Contaminant Monitoring Rule List 3 (U.S. EPA 2006).

Exposure to chemical contaminants can occur in both private wells and community water supplies, and may present health risks. High nitrate levels in water used in mixing infant formula have been associated with risk for methemoglobinemia (blue-baby syndrome) in infants under 6 months of age, although other health factors such as diarrhea and respiratory disease have also been implicated (Ward et al. 2005). The U.S. EPA drinking water standard of 10 mg/L NO_3_–N and the WHO guideline of 11 mg/L NO_3_–N were set because of concerns about methemo-globinemia. (Note: “nitrate” refers to nitrate–nitrogen). Epidemiologic studies of noncancer health outcomes and high nitrate levels in drinking water have reported an increased risk of hyperthyroidism (Seffner 1995) from long-term exposure to levels between 11–61 mg/L (Tajtakova et al. 2006). Drinking water nitrate at levels < 10 mg/L has been associated with insulin-dependent diabetes (IDDM; Kostraba et al. 1992), whereas other studies have shown an association with IDDM at nitrate levels > 15 mg/L (Parslow et al. 1997) and > 25 mg/L (van Maanen et al. 2000). Increased risks for adverse reproductive outcomes, including central nervous system malformations (Arbuckle et al. 1988) and neural tube defects (Brender et al. 2004; Croen et al. 2001), have been reported for drinking water nitrate levels < 10 mg/L.

Anecdotal reports of reproductive effects of nitrate in drinking water include a case study of spontaneous abortions in women consuming high nitrate water (19–26 mg/L) from private wells (Morbidity and Mortality Weekly Report 1996).

While amassing experimental data suggest a role for nitrate in the formation of carcinogenic *N*-nitroso compounds, clear epidemio-logic findings are lacking on the possible association of nitrate in drinking water with cancer risk. Ecologic studies have reported mixed results for cancers of the stomach, bladder, and esophagus (Barrett et al. 1998; Cantor 1997; Eicholzer and Gutzwiller 1990; Morales-Suarez-Varela et al. 1993, 1995) and non-Hodgkin lymphoma (Jensen 1982; Weisenburger 1993), positive findings for cancers of the nasopharynx (Cantor 1997), prostate (Cantor 1997), uterus (Jensen 1982; Thouez et al. 1981), and brain (Barrett et al. 1998), and negative findings for ovarian cancer (Jensen 1982; Thouez et al. 1981). Positive findings have generally been for long-term exposures at > 10 mg/L nitrate. Case–control studies have reported mixed results for stomach cancer (Cuello et al. 1976; Rademacher et al. 1992; Yang et al. 1998); positive results for non-Hodgkin lymphoma at > 4 mg/L nitrate (Ward et al. 1996) and colon cancer at > 5 mg/L (De Roos et al. 2003); and negative results for cancers of the brain (Mueller et al. 2001; Steindorf et al. 1994), bladder (Ward et al. 2003), and rectum (De Roos et al. 2003), all at < 10 mg/L. Cohort studies have reported no association between nitrate in drinking water and stomach cancer (Van Loon et al. 1998); positive associations with cancers of the bladder and ovary at long-term exposures > 2.5 mg/L (Weyer et al. 2001); and inverse associations with cancers of the rectum and uterus, again at > 2.5 mg/L (Weyer et al. 2001).

Exposure to low levels of antibiotics and other pharmaceuticals in drinking water (generally at micrograms per liter or nanograms per liter) represent unintentional doses of substances generally used for medical purposes to treat active disease or prevent disease. The concern is more related to possible cumulative effects of long-term low-dose exposures than on acute health effects (Daughton and Ternes 1999). A recent study conducted in Germany found that the margin between indirect daily exposure via drinking water and daily therapeutic dose was at least three orders of magnitude, concluding that exposure to pharmaceuticals via drinking water is not a major health concern (Webb et al. 2003). It should be noted that when prescribing medications, providers ensure patients are not taking incompatible drugs, but exposure via drinking water is beyond their control.

Endocrine-disrupting compounds are chemicals that exhibit biological hormonal activity, either by mimicking natural estrogens, by canceling or blocking hormonal actions, or by altering how natural hormones and their protein receptors are made (McLachlan and Korach 1995). Although very low levels of estrogenic compounds can stimulate cell activity, the potential for human health effects, such as breast and prostate cancers, and reproductive effects from exposure to endocrine disruptors, is in debate (Weyer and Riley 2001).

## Workshop Recommendations

### Priority research needs

- Ecosystems monitoring: Systematic sustained studies of ecosystem health in proximity to large CAFOs are needed, including effects of input spikes during spills or flooding events.
- Toxicologic assessment of contaminants: Identification and prioritization of contaminants are needed to identify those that are most significant to environmental and public health. Toxicity studies need to be conducted to identify and quantify contaminants (including metabolites), and to investigate interactions (synergistic, additive, and antagonistic effects).
- Fate and transport: Studies of parent compounds and metabolites in soil and water must be conducted, and the role of sediment as a carrier and reservoir of contaminants must be evaluated.
- Surveillance programs: Programs should be instituted to assess private well water quality in high-risk areas. Biomonitoring programs should be designed and implemented to assess actual dose from environmental exposures.

### Translation of science to policy

- Wastewater and drinking water treatment: Processes for water treatment must be monitored to ensure adequate removal or inactivation of emerging contaminants.
- Pollution prevention: Best management practices should be implemented to prevent or minimize release of contaminants into the environment.
- Education: Educational materials should be continued to be developed and distributed to agricultural producers.

## References

[b1-ehp0115-000308] Aneja VP, Nelson DR, Roelle PA, Walker JT (2003). Agricultural ammonia emissions and ammonium concentrations associated with aerosols and precipitation in the southeast United States. J Geophys Res.

[b2-ehp0115-000308] Arbuckle TE, Sherman GJ, Corey PN, Walters D, Lo B (1988). Water nitrates and CNS birth defects: a population-based case-control study. Arch Environ Health.

[b3-ehp0115-000308] Barker JC Zublena JP 1995. Livestock Manure Nutrient Assessment in North Carolina. Final Report. Raleigh, NC: North Carolina Agricultural Extension Service, North Carolina State University.

[b4-ehp0115-000308] Barnes KK, Christenson SC, Kolpin DW, Focazio MJ, Furlong ET, Zaugg SD (2004). Pharmaceuticals and other organic wastewater contaminants within a leachate plume downgradient of a municipal landfill. Ground Water Monitoring Rev.

[b5-ehp0115-000308] Barrett JH, Parslow RC, McKinney PA, Law GR, Forman D (1998). Nitrate in drinking water and the incidence of gastric, esophageal, and brain cancer in Yorkshire, England. Cancer Causes Control.

[b6-ehp0115-000308] Boxall ABA, Kolpin DW, Halling-Sorenson B, Tolls J (2003). Are veterinary medicines causing environmental risks?. Environ Sci Technol.

[b7-ehp0115-000308] Boxall ABA, Sinclair CJ, Fenner K, Kolpin DW, Maund SJ (2004). When synthetic chemicals degrade in the environment. Environ Sci Technol.

[b8-ehp0115-000308] Brain RA, Wilson CJ, Johnson DJ, Sanderson H, Bestari K, Hanson ML (2005). Effects of a mixture of tetracy-clines to *Lemna gibba* and *Miriophyllum sibiricum* evaluated in aquatic microcosms. Environ Pollution.

[b9-ehp0115-000308] Brender JD, Olive JM, Felkner M, Suarez L, Marckwardt W, Hendricks KA (2004). Dietary nitrites and nitrates, nitros-able drugs, and neural tube defects. Epidemiology.

[b10-ehp0115-000308] Burkholder JM, Mallin MA, Glasgow HB, Larsen LM, McIver MR, Shank GC (1997). Impacts to a coastal river and estuary from rupture of a large swine waste holding lagoon. J Environ Qual.

[b11-ehp0115-000308] Burkholder JM, Mason KM, Glasgow HB (1992). Water-column nitrate enrichment promotes decline of eelgrass (*Zostera marina* L.): evidence from seasonal mesocosm experiments. Mar Ecol Prog Ser.

[b12-ehp0115-000308] Burnison BK, Hartmann A, Lister A, Servos MR, Ternes T, Van der Kraak G (2003). A toxicity identification evaluation approach to studying estrogenic substances in hog manure and agricultural runoff. Environ Toxicol Chem.

[b13-ehp0115-000308] Campagnolo ER, Johnson KR, Karpati A, Rubin CS, Kolpin DW, Meyer MT (2002). Antimicrobial residues in animal waste and water resources proximal to large-scale swine and poultry feeding operations. Sci Total Environ.

[b14-ehp0115-000308] Cantor KP (1997). Drinking water and cancer. Cancer Causes Control.

[b15-ehp0115-000308] Carmichael WW, Azevedo SMFO, An JS, Molica RJR, Jochimsen EM, Lau S (2001). Human fatalities from cyanobacteria: chemical and biological evidence for cyanotoxins. Environ Health Perspect.

[b16-ehp0115-000308] Chorus I Bartram J eds. 1999. Toxic Cyanobacteria in Water—A Guide to their Public Health Consequences, Monitoring and Management. Geneva:World Health Organization. Available: http://www.who.int/water_sanitation_health/resourcesquality/toxicyanbact/en/ [accessed 5 January 2007].

[b17-ehp0115-000308] Christian T, Schneider RJ, Barber HA, Skutlarek D, Meyer GT, Goldrach HE (2003). Determination of antibiotic residues in manure, soil, and surface waters. Acta Hydrochim Hydrobiol.

[b18-ehp0115-000308] Cordy G, Duran N, Bower H, Rice R, Kolpin DW, Furlong ET (2004). Do pharmaceuticals, pathogens, and other organic wastewater compounds persist when wastewater is used for recharge?. Ground Water Monitoring Rev.

[b19-ehp0115-000308] Costanzo SD, Murby J, Bates J (2005). Ecosystem response to antibiotics entering the aquatic environment. Marine Pollut Bull.

[b20-ehp0115-000308] Croen LA, Todoroff K, Shaw GM (2001). Maternal exposure to nitrate from drinking water and diet and risk for neural tube defects. Am J Epidemiol.

[b21-ehp0115-000308] Cromwell GL (2002). Why and how antibiotics are used in swine production. Anim Biotechnol.

[b22-ehp0115-000308] Cuello C, Correa P, Haenszel W, Gordillo G, Brown C, Archer M (1976). Gastric cancer in Columbia. 1. Cancer risk and suspect environmental agents. J Natl Cancer Inst.

[b23-ehp0115-000308] Daughton CG, Ternes TA (1999). Pharmaceuticals and personal care products in the environment: agents of subtle change?. Environ Health Perspect.

[b24-ehp0115-000308] Delepee R, Pouliquen H, Le Bris H (2004). The bryophyte *Fontinalis antipyretica* Hedw. bioaccumulates oxytetracycline, flumequine and oxolinic acid in the freshwater environment. Sci Total Environ.

[b25-ehp0115-000308] De Roos AJ, Ward MH, Lynch CF, Cantor KP (2003). Nitrate in public water supplies and the risk of colon and rectum cancers. Epidemiology.

[b26-ehp0115-000308] Dietze JE, Scribner EA, Meyer MT, Kolpin DW (2005). Occurrence of antibiotics in water from 13 fish hatcheries, 2001–03. Intern J Environ Anal Chem.

[b27-ehp0115-000308] Eicholzer M, Gutzwiller F (1990). Dietary nitrates, nitrites and *N*-nitroso compounds and cancer risk: a review of the epi-demiologic evidence. Nutr Rev.

[b28-ehp0115-000308] Evans RO, Westerman PW, Overcash MR (1984). Subsurface drainage water quality from land application of swine lagoon effluent. Trans Am Soc Agric Eng.

[b29-ehp0115-000308] Extension Toxicology Network 2003. Exotoxnet—A Pesticide Information Project of the Cooperative Extension Offices of Cornell University, Michigan State University, Oregon State University, and the University of California at Davis. U.S. Department of Agriculture, Extension Service, and the National Agricultural Pesticide Impact Assessment Program. Available: http://ace.orst.edu/info/extoxnet/ [accessed 26 September 2005].

[b30-ehp0115-000308] Fernandez C, Alonso C, Babin MM, Pro J, Carbonell G, Tarazona JV (2004). Ecotoxicological assessment of doxy-cycline in aged pig manure using multispecies soil systems. Sci Total Environ.

[b31-ehp0115-000308] Gaskins HR, Collier CT, Anderson DB (2002). Antibiotics as growth promoters: mode of action. Anim Biotechnol.

[b32-ehp0115-000308] Gerba CP, Smith JE (2005). Sources of pathogenic microor-ganisms and their fate during land application of wastes. J Environ Qual.

[b33-ehp0115-000308] Glassmeyer ST, Furlong ET, Kolpin DW, Cahill JD, Werner SL, Meyer MT (2005). Transport of chemical and microbial contaminants from known wastewater discharges: Potential for use as indicators of human fecal contamination. Environ Sci Technol.

[b34-ehp0115-000308] Halling-Sorensen B, Sengelov G, Ingerslev F, Jensen LB (2003). Reduced antimicrobial potencies of oxytetracycline, tylosin, sulfadiazine, streptomycin, ciprofloxacin, and olaquindox due to environmental processes. Arch Environ Contam Toxicol.

[b35-ehp0115-000308] Ham JM, DeSutter TM (2000). Toward site-specific design standards for animal-waste lagoons: protecting ground water quality. J Environ Qual.

[b36-ehp0115-000308] Hanselman TA, Graetz DA, WIlkie AC (2003). Manure-borne estrogens as potential environmental contaminants: a review. Environ Sci Technol.

[b37-ehp0115-000308] Hirsch R, Ternes T, Haberer K, Kratz KL (1999). Occurrence of antibiotics in the aquatic environment. Sci Total Environ.

[b38-ehp0115-000308] Hitzfield BC, Hoger SJ, Dietrich DR (2000). Cyanobacterial toxins: Removal during drinking water treatment, and human risk assessment. Environ Health Perspect.

[b39-ehp0115-000308] Huddleston JH 1996. How Soil Properties Affect Groundwater Vulnerability to Pesticides Contamination. Oregon State Extension Service. Available: http://wwwagcomm.ads.orst.edu/AgComWebFile/EdMat/EM8559.pdf [accessed 26 September 2005].

[b40-ehp0115-000308] Huffman RL, Westerman PW (1995). Estimated seepage losses from established swine waste lagoons in the lower coastal plain of North Carolina. Transact Am Soc Agric Eng.

[b41-ehp0115-000308] Jensen K, Krogh PH, Sverdup LE (2003). Effects of the antibacterial agents tiamulin, olanquindox and metronidazole and the antihelminthic ivermectin on the soil invertebrate species *Folsomia fimeteria* (Collembola) and *Enchytraeus crypticus* (Enchytraeidae). Chemosphere.

[b42-ehp0115-000308] Jensen OM (1982). Nitrate in drinking water and cancer in northern Jutland, Denmark, with special reference to stomach cancer. Ecotoxicol Environ Saf.

[b43-ehp0115-000308] Jongbloed AW, Lenis NP (1998). Environmental concerns about animal manure. J Anim Sci.

[b44-ehp0115-000308] Khachatourians GG (1998). Agricultural use of antibiotics and the evolution and transfer of antibiotic-resistant bacteria. Can Med Assoc J.

[b45-ehp0115-000308] Kolpin DW, Furlong ET, Meyer MT, Thurman EM, Zaugg SD, Barber LB (2002). Pharmaceuticals, hormones and other organic wastewater contaminants in U.S. streams, 1999–2000: a national reconnaissance. Environ Sci Technol.

[b46-ehp0115-000308] Kostraba JN, Gay EC, Rewers M, Hamman RF (1992). Nitrate levels in community drinking waters and risk of IDDM: an ecological analysis. Diabetes Care.

[b47-ehp0115-000308] Kummerer K (2004). Resistance in the environment. J Antimicrob Chemother.

[b48-ehp0115-000308] Liu X, Miller GY, McNamara PE (2005). Do antibiotics reduce production risk for U.S. pork producers?. J Agric Appl Econ.

[b49-ehp0115-000308] Mallin MA (2000). Impacts of industrial-scale swine and poultry production on rivers and estuaries. Am Sci.

[b50-ehp0115-000308] Mallin MA, Burkholder JM, Shank GC, McIver MR, Glasgow HB, Springer J (1997). Comparative impacts of effluent from poultry and swine waste holding lagoon spills on receiving rivers and tidal creeks. J Environ Qual.

[b51-ehp0115-000308] McGechan MB, Lewis DR, Hooda PS (2005). Modelling through-soil transport of phosphorous to surface waters from livestock agriculture at the field and catchment scale. Sci Total Environ.

[b52-ehp0115-000308] McLachlan JA, Korach KS (1995). Symposium on Estrogens in the Environment, III. Environ Health Perspect.

[b53-ehp0115-000308] Mellon MC Benbrook C Benbrook KL 2001. Estimates of antimicrobial abuse in livestock. Cambridge, MA:Union of Concerned Scientists.

[b54-ehp0115-000308] Meyer MT 2004. Use and Environmental Occurrence of Veterinary Pharmaceuticals in the United States. In: Pharmaceuticals in the Environment: Sources, Fate, Effects, and Risks (Kummerer K, ed). New York:Springer-Verlag,155–163.

[b55-ehp0115-000308] Morales-Suarez-Varela M, Llopis-Gonzales A, Tejerizo-Perez ML, Ferrandiz Ferragud J (1993). Concentration of nitrates in drinking water and its relationship with bladder cancer. J Environ Pathol Toxicol Oncol.

[b56-ehp0115-000308] Morales-Suarez-Varela MM, Llopis-Gonzalez A, Tejerizo-Perez ML (1995). Impact of nitrates in drinking water on cancer mortality in Valencia, Spain. Eur J Epidemiol.

[b57-ehp0115-000308] Morbidity and Mortality Weekly Report (MMWR) (1996). Spontaneous abortions possibly related to ingestion of nitrate-contaminated well water—LaGrange County, Indiana, 1991–1994. MMWR.

[b58-ehp0115-000308] Mueller BA, Newton K, Holly EA, Preston-Martin S (2001). Residential water source and the risk of childhood brain tumors. Environ Health Perspect.

[b59-ehp0115-000308] Mueller DK Hamilton PA Helsel DR Hitt KJ Ruddy BC 1995. Nutrients in groundwater and surface water of the United States—an analysis of data through 1992. US Geological Survey Water Resour Invest Rep 95–4031.

[b60-ehp0115-000308] Parslow RC, McKinney PA, Law GR, Staines A, Williams B, Bodansky HJ (1997). Incidence of childhood diabetes mellitus in Yorkshire, northern England, is associated with nitrate in drinking water: an ecological analysis. Diabetologia.

[b61-ehp0115-000308] Rademacher JJ, Young TB, Kanarek MS (1992). Gastric cancer mortality and nitrate levels in Wisconsin drinking water. Arch Environ Health.

[b62-ehp0115-000308] Raman DR, Williams EL, Layton AC, Burns RT, Easter JP, Daugherty AS (2004). Estrogen content of dairy and swine wastes. Environ Sci Technol.

[b63-ehp0115-000308] Rao PV, Gupta N, Bhaskar AS, Jayaraj R (2002). Toxins and bioactive compounds from cyanobacteria and their implications on human health. J Environ Biol.

[b64-ehp0115-000308] Rapala J, Lahti K, Rasanen LA, Esala AL, Niemela SI, Sivonen K (2002). Endotoxins associated with cyanobacteria and their removal during drinking water treatment. Water Res.

[b65-ehp0115-000308] Schets FM, During M, Italiaander R, Heijnen L, Rutjes SA, van der Zwaluw WK (2005). *Escherichia coli* O157:H7 in drinking water from private water supplies in the Netherlands. Water Res.

[b66-ehp0115-000308] Seffner W (1995). Natural water contents and endemic goiter. Zantralblatt Hyg Umwelt.

[b67-ehp0115-000308] Sengelov G, Agerso Y, Halling-Sorensen B, Baloda SB, Andersen JS, Jensen LB (2003). Bacterial antibiotic resistance levels in Danish farmland as a result of treatment with pig manure slurry. Environ Int.

[b68-ehp0115-000308] Sharpe RR, Harper LA (1997). Ammonia and nitrous oxide emissions from sprinkler irrigation applications of swine effluent. J Environ Qual.

[b69-ehp0115-000308] Sharpley A, Moyer B (2000). Phosphorus forms in manure and compost and their release during simulated rainfall. J Environ Qual.

[b70-ehp0115-000308] Soto AM, Calabro JM, Prechtl NV, Yau AY, Orlando EF, Daxenberger A (2004). Androgenic and estrogenic activity in water bodies receiving cattle feedlot effluent in eastern Nebraska, USA. Environ Health Perspect.

[b71-ehp0115-000308] Steindorf K, Schlehofer B, Becher H, Hornig G, Wahrendorf J (1994). Nitrate in drinking water: a case-control study on primary brain tumours with an embedded drinking water survey in Germany. Int J Epidemiol.

[b72-ehp0115-000308] Stone KC, Hunt PG, Coffey SW, Matheny TA (1995). Water quality status of A USDA water quality demonstration project in the Eastern Coastal Plain. J Soil Wat Conserv.

[b73-ehp0115-000308] Sumpter JP, Johnston AC (2005). Lessons from endocrine disruption and their application to other issues concerning trace organics in the aquatic environment. Environ Sci Technol.

[b74-ehp0115-000308] Tajtakova M, Semanova Z, Tomkova Z, Szokeova E, Majoroa J, Radikova Z (2006). Increased thyroid volume and frequency of thyroid disorders signs in schoolchildren from nitrate polluted area. Chemosphere.

[b75-ehp0115-000308] Thiele-Bruhn S, Beck IC (2005). Effects of sulfonamide and tetracycline antibiotics on soil microbial activity and microbial biomass. Chemosphere.

[b76-ehp0115-000308] Thouez J-P, Beauchamp Y, Simard A (1981). Cancer and the physicochemical quality of drinking water in Quebec. Soc Sci Med.

[b77-ehp0115-000308] University of Iowa and Iowa State Study Group 2002. Iowa Concentrated Animal Feeding Operations Air Quality Study. Iowa City, IA:The University of Iowa College of Public Health.

[b78-ehp0115-000308] U.S. EPA 1998. Environmental Impacts of Animal Feeding Operations. Washington, DC:U.S. Environmental Protection Agency, Office of Water, Standards and Applied Sciences Division. Available: http://www.epa.gov/ostwater/guide/feedlots/envimpct.pdf [accessed 26 September 2005].

[b79-ehp0115-000308] U.S. EPA 2000. Deposition of Air Pollutants to the Great Waters. 3rd Report to the U.S. Congress. (1) Section A. Washington, DC:U.S. Environmental Protection Agency.

[b80-ehp0115-000308] U.S. EPA 2006. Approved Methods for Unregulated Contaminants. U.S. Environmental Protection Agency. Available: http://www.epa.gov/ogwdw/methods/unregtbl.html [accessed 5 January 2007].

[b81-ehp0115-000308] Van Loon AJM, Botterweck AAM, Goldbohm RA, Brants HAM, van Klaveren JD, van den Brandt PA (1998). Intake of nitrate and nitrite and the risk of gastric cancer: a prospective cohort study. Br J Cancer.

[b82-ehp0115-000308] Van Maanen JM, Albering HJ, de Kok TM, van Breda SG, Curfs DM, Vermeer IT (2000). Does the risk of childhood diabetes mellitus require revision of the guideline values for nitrate in drinking water?. Environ Health Perspect.

[b83-ehp0115-000308] Ward MH, Cantor KP, Riley D, Merkle S, Lynch CF (2003). Nitrate in public water supplies and risk of bladder cancer. Epidemiology.

[b84-ehp0115-000308] Ward MH, deKok TM, Levallois P, Brender J, Gulis G, Nolan BT (2005). Workgroup report: drinking-water nitrate and health—recent findings and research needs. Environ Health Perspect.

[b85-ehp0115-000308] Ward MH, Mark SD, Cantor KP, Weisenburger DD, Correa-Villasenore A, Zahm SH (1996). Drinking water and the risk of non-Hodgkin’s lymphoma. Epidemiology.

[b86-ehp0115-000308] Webb J Archer JR 1994. Pollution of soils and watercourses by wastes from livestock production systems. In: Pollution in Livestock Production Systems (Dewi IA, Axford RFE, Marai IFM, Omed HM, eds). Oxfordshire, UK:CABI Publishing, 189–204.

[b87-ehp0115-000308] Webb S, Ternes T, Gibert M, Olejniczak K (2003). Indirect human exposure to pharmaceuticals via drinking water. Toxicol Lett.

[b88-ehp0115-000308] Weisenburger D (1993). Potential health consequences of ground-water contamination of nitrates in Nebraska. Nebr Med J.

[b89-ehp0115-000308] Westerman PW, Huffman RL, Feng JS (1995). Swine-lagoon seepage in sandy soil. Transact ASAE.

[b90-ehp0115-000308] Weyer P Riley D 2001. Endocrine Disruptors and Pharmaceuticals in Drinking Water. Denver, CO:AWWA Research Foundation and the American Water Works Association.

[b91-ehp0115-000308] Weyer PJ, Cerhan JR, Kross BC, Hallberg GR, Kantamneni J, Breuer G (2001). Municipal drinking water nitrate level and cancer risk in older women: the Iowa Women’s Health Study. Epidemiology.

[b92-ehp0115-000308] WHO 2003. Algae and cyanobacteria in fresh water. In: Guidelines for Safe Recreational Water Environments. Vol 1: Coastal and Fresh Waters. Geneva:World Health Organization, 136–138.

[b93-ehp0115-000308] Wing S, Freedman S, Band L (2002). The potential impact of flooding on confined animal feeding operations in eastern North Carolina. Environ Health Perspect.

[b94-ehp0115-000308] Wollenberger L, Halling-Sorensen B, Kusk KO (2000). Acute and chronic toxicity of veterinary antibiotics to *Daphnia magna*. Chemosphere.

[b95-ehp0115-000308] Yang C-Y, Cheng M-F, Tsai S-S, Hsieh Y-L (1998). Calcium, magnesium, and nitrate in drinking water and gastric cancer mortality. Jpn J Cancer Res.

[b96-ehp0115-000308] Zahn JA, Hatfield JL, Do YS (1997). Characterization of volatile organic emissions and wastes from a swine production facility. J Environ Qual.

